# HIF-2α accumulation in human monocytes upon transfer of hypoxia-associated miRNAs via plasma-derived small extracellular vesicles from head and neck cancer patients

**DOI:** 10.3389/fonc.2025.1701388

**Published:** 2026-01-12

**Authors:** Marie-Nicole Theodoraki, Linda Hofmann, Helen Bauer, Jonas Fleckner, Reinhard Depping, Christian Idel, Kirstin Plötze-Martin, Zuzana Penxova, Dirk Rades, Thomas K. Hoffmann, Karl-Ludwig Bruchhage, Ralph Pries

**Affiliations:** 1 Department of Otorhinolaryngology, Ulm University Medical Center, Ulm, Germany; 2 Department of Otolaryngology, Head and Neck Surgery, School of Medicine and Health, Technical University of Munich (TUM), Munich, Germany; 3 Department of Otorhinolaryngology, University of Luebeck, Luebeck, Germany; 4 Institute of Physiology, Working Group Hypoxia, University of Luebeck, Luebeck, Germany; 5 Department of Radiation Oncology, University of Luebeck, Luebeck, Germany

**Keywords:** HIF-2α, HNSCC, hypoxia, liquid biomarker, miRNA, monocytes, small extracellular vesicles

## Abstract

Hypoxia is an important hallmark of the tumor microenvironment (TME) in solid tumors and is closely associated with resistance to radiotherapy and a poorer clinical outcome. Tumor-associated plasma-derived small extracellular vesicles (sEVs) have gained increasing attention as an important regulatory TME component for tumor progression and immune evasion. This study aimed to investigate the involvement of plasma-derived sEVs from head and neck squamous cell carcinoma (HNSCC) patients in the systemic regulation of hypoxia-related molecular pathways in circulating immune cells. Plasma-derived sEVs of healthy donors (HDs) and HNSCC patients were isolated and evaluated for morphology, size, miRNA cargo composition, and their influence on monocyte characteristics. Transfer of plasma-derived sEVs from HNSCC patients stimulated increased levels of checkpoint molecule PD-L1 and chemokine CXCL4 secretion. An accumulation of hypoxia-inducible factor (HIF)-2α was associated with hypoxia-regulating miRNAs in sEVs from HNSCC patients. This provides new insights into a proposed tumor-associated systemic sEV-miRNA-mediated hypoxia transfer.

## Introduction

Head and neck squamous cell carcinoma (HNSCC) is a heterogeneous malignant disease arising from the mucosal epithelium in the oral cavity, pharynx, and larynx (1, 2). The poor prognosis of patients with recurrent or metastatic HNSCC underlines the urgent need for innovative prognostic biomarkers to predict the individual course of disease and to enable promising treatment decisions (3).

In this context, tumor-associated plasma-derived small extracellular vesicles (sEVs) have gained increasing attention as circulating liquid indicators for HNSCC disease stage and tumor progression (4–7). sEVs are secreted by the vast majority of cells and present an important component of systemic intercellular communication by transferring their cargo, including proteins and nucleic acids, between donor and recipient cells (8–12). Different studies have revealed that intercellular sEV transfer is the main way of sEV-mediated modulation of recipient cells (13, 14). Our recent data revealed significantly increased secretion patterns of chemokine (C-X-C motif) ligand 4 (CXCL4; platelet factor 4) by monocyte-derived macrophages in response to the internalization of plasma-derived sEVs from HNSCC patients (15). CXCL4 is known to promote monocyte adhesion to the endothelial wall of the blood vessels and thus to support their extravasation into the tissue (16, 17).

It has recently been shown that hypoxia is a fundamental driver of CXCL4 secretion by umbilical cord CD34^+^ cell-derived plasmacytoid dendritic cells via an overproduction of mitochondrial reactive oxygen species (mtROS) and the stabilization of hypoxia-inducible factor (HIF)-2α (18). Accordingly, increased CXCL4 secretion levels by THP-1 monocytes (Tohoku Hospital Pediatrics-1) (19) were also observed in response to hypoxic growth conditions (20). Furthermore, measurements in patients suffering from obstructive sleep apnea syndrome (OSAS) revealed significant redistributions of peripheral blood monocyte subsets and increased monocytic expression of T cell-inhibiting checkpoint molecule programmed death ligand-1 (PD-L1; CD274) due to the related hypoxia-driven underlying systemic inflammation (21).

Hypoxia is a hallmark of the microenvironment in solid tumors and is closely associated with resistance to radiotherapy (RT) and a poorer clinical outcome. Hypoxia is a major cellular stress factor, and it is well established that hypoxia-related factors affect different innate immunity pathways (22) and also influence the size and quantity of sEVs and the composition of the sEV cargos (23). However, the involvement of plasma-derived sEVs from HNSCC patients in the systemic regulation of hypoxia-related molecular pathways in innate immune cells has not been studied so far.

Therefore, cells of the human monocyte leukemia cell line THP-1 were incubated with plasma-derived sEVs from healthy donors and HNSCC patients before and after radio/chemotherapeutic treatment. Recent data revealed a partial recovery of peripheral blood monocyte subsets in response to radio(chemo)therapy of head and neck cancer patients (24). Monocytes were analyzed by flow cytometry concerning the surface expression levels of adhesion molecules CD11a (integrin-α L; LFA-1), CD11b (integrin-α M; Mac-1), CD11c (integrin-α X), CX3CR1 (CX3CL1 receptor), and checkpoint molecule PD-L1 (CD274; programmed cell death ligand-1), all of which are known to be differentially regulated in distinct environmental conditions (25). Furthermore, the monocytic secretion of chemokine CXCL4 and the expression of HIF-1α and HIF-2α in monocytes were analyzed with regard to the influence of plasma-derived sEVs. Finally, profiling of hypoxia-associated sEV miRNAs was performed based on a larger cohort of HNSCC patients and healthy donors to ensure a solid data situation of these sensitive liquid biomarkers.

The study aimed to elucidate a possible “hypoxia transfer” via HNSCC plasma-derived sEVs to innate immune cells and their associated immunological consequences.

## Materials and methods

### Ethics statement

Patients were examined at the Clinic of Otorhinolaryngology, University Hospital Schleswig-Holstein, Campus Luebeck, after written informed consent was obtained. The study was conducted in accordance with the ethical approval of the local ethics committee at the University of Luebeck (approval number 16-278) and the World Medical Association (WMA) Declaration of Helsinki.

### Isolation of sEVs from plasma

Plasma-derived sEVs were isolated by size exclusion chromatography (SEC) (for characterization and functional assays) or ultracentrifugation (for miRNA analysis) as described previously (26, 27). In short, thawed plasma was pre-cleared by centrifugation at 2,000 × *g* for 10 min and 12,000 × *g* for 30 min at 4°C, followed by filtration through 0.22-µm syringe-driven filters (Millipore, Burlington, MA, USA). For size exclusion chromatography, 1-mL aliquots were loaded on pre-packed Sepharose columns and sequentially eluted with 1 mL phosphate-buffered saline (PBS). Fraction #4, shown to contain biologically and morphologically intact single vesicles (26), was collected and used for characterization and functional analyses. For ultracentrifugation, pre-cleared plasma was diluted 1:1 with PBS and centrifuged at 110,000 × *g* for 2 hours at 4°C. Supernatants were removed, and the sEV pellet was resuspended in 1 mL PBS and centrifuged at 110,000 × *g* for 70 min at 4°C. After repeating this washing step, the sEV pellet was dissolved in 700 µL QIAzol Lysis Reagent (Qiagen, Hilden, Germany) and preserved at −80°C until processing.

Pre-clearance of plasma by low-speed centrifugation and ultrafiltration, paired with two subsequent washes of the UC pellet and QIAzol-based phase separation during RNA extraction, reduces contamination with non-EV RNA (ribonucleoprotein complexes, free nucleic acids).

### Blood collection and patient data

Blood samples from healthy donors (HDs; n = 10; mean age of 61.5; three women and seven men) and HNSCC patients (n = 15; mean age of 62; four female and 11 men) were obtained by venipuncture into a sodium citrate-containing S-Monovette (Sarstedt, Nümbrecht, Germany). After centrifugation at 1,000 × *g* for 10 min, plasma samples were preserved at −80°C for later investigations. The clinicopathological characteristics of patients are listed in **Table 1**.

**Table 1:** Clinicopathological parameters.

Characteristics	Patients (n = 15)	
	n	%
Gender		
M	11	72
F	4	28
Tumor site		
Pharynx	9	61
Larynx	2	14
Oral cavity	4	27
Tumor stage		
T1–T2	6	39
T3–T4	9	61
Human papillomavirus (HPV) status		
Positive	11	72
Negative	2	14
Unknown	2	14
Alcohol abuse		
Yes	3	22
No	12	78
Tobacco consumption		
Yes	13	86
No	2	14

### Characterization of sEVs

To estimate sEV quantity and to adjust the concentration for functional assays, the total sEV protein concentration of the obtained fraction #4 was measured using Pierce BCA Protein Assay (Thermo Fisher Scientific, Waltham, MA, USA) according to the manufacturer’s instructions. Briefly, 10 µL of standard or sample and 200 µL reagent mix (A:B ratio 50:1) were added to a transparent, flat-bottom 96-well plate. The plate was incubated at 37°C for 30 min, and absorption at 562 nm was measured using a TECAN plate reader. Protein concentration was calculated based on the obtained standard curve.

sEVs were concentrated using 100-kDa cutoff centrifugal filters (Millipore, Burlington, MA, USA). sEVs were characterized in line with the minimal information for studies of extracellular vesicles (MISEV) 2023 recommendations (28) as described in detail in our previous publication (7) (EV-Track ID EV200068). In summary, Western blotting for the detection of the endosomal protein TSG101 and the sEV-associated tetraspanins CD9 and CD63 was performed to verify the origin of the vesicles. Transmission electron microscopy (TEM) and nanoparticle tracking analysis (NTA) were used for the evaluation of morphology and size, and particle counting, respectively.

### MiRNA profiling of sEVs

Profiling of sEV miRNA was performed as previously described (27). In short, RNA was isolated using the miRNeasy Micro Kit (Qiagen, Hilden, Germany) according to the manufacturer’s instructions. Quality and quantity were determined using a High Sensitivity RNA Screen Tape on a 4150 TapeStation (Agilent Technologies, Santa Clara, CA, USA) and the microRNA assay on a Qubit Fluorometer (Thermo Fisher Scientific, Waltham, MA, USA), respectively. sEV RNA was stored at −80°C. The Human v3 miRNA Assay, comprising multiplexed analysis of 827 human miRNAs, was performed using 500 pg of sEV RNA on the nCounter^®^ SPRINT system (Nanostring Technologies, Seattle, WA, USA) at the nCounter^®^ Core Facility of the University of Heidelberg, Germany. MiRNA expression data were analyzed using the nSolver 4.0 software. Raw data (counts of individual miRNAs) were normalized based on the positive ligation controls and shown in **Supplementary Table 1**. For better readability, “hsa” was removed from the miRNA names throughout the manuscript.

### Cell culture and incubation conditions

For cell culture experiments, the non-adherent monocyte cell line THP-1 (Tohoku Hospital Pediatrics-1) was used. Cell culture was performed in Roswell Park Memorial Institute (RPMI) 1640 medium supplemented with 10% heat-inactivated fetal bovine serum (FBS; Thermo Fisher Scientific, Waltham, MA, USA; Cat: A5256701), 1% sodium pyruvate, and 1% streptomycin/penicillin at 37°C and 5% CO_2_ under a humidified atmosphere. Cells were subcultured every 3 days at a maximum density of 1 × 10^6^ cells/mL. Hypoxic conditions of 5% O_2_ were induced by culturing the cells for 24 hours at 37°C in a humidified incubator (Heracell Vios 160i CO_2_ Incubator, Thermo Fisher Scientific, Waltham, MA, USA). To analyze the influence of plasma-derived sEVs from HNSCC patients or healthy donors, THP-1 cells were incubated with 10 µg of isolated sEVs/mL for 24 hours.

### Cytokine analysis

Cell culture supernatants were collected, instantly frozen with liquid nitrogen, and preserved at −80°C. Secretion levels of chemokine CXCL4 were determined via enzyme-linked immunosorbent assay (ELISA) according to the manufacturer’s protocols (R&D Systems, Minneapolis, MN, USA; Cat: DPF40). In brief, a monoclonal antibody specific for human CXCL4 was pre-coated onto a microplate. The supernatants of monocytes were added to the well plate for incubation. After discarding the solution, 200 μL of biotinylated antibody was added to each well. Following rinsing the well plate, the enzyme conjugate working solution and substrate were added to the wells. After terminating the reaction, the optical density at 450 nm was measured.

### Immunoblot analysis

Cells were washed with ice-cold PBS (Thermo Fisher Scientific, Waltham, MA, USA; Cat: A5256701), and protein extraction was carried out using reagents purchased by Thermo Fisher (Waltham, MA, USA) according to the manufacturer’s protocol.

Protein concentration was determined using Bio-Rad DC Protein Assay (Bio-Rad, Hercules, CA, USA). Proteins (30 µg) were separated via Sodium Dodecyl Sulfate Polyacrylamide Gel Electrophoresis (SDS–PAGE) and transferred onto polyvinylidene difluoride membrane (PVDF; Merck Millipore, Burlington, MA, USA) by semi-dry electroblotting. The blots were incubated with primary antibodies for 24 hours and with Horseradish Peroxidase (HRP)-conjugated secondary antibodies (Dako Denmark, Glostrup, Denmark; Cat: K4065) in a concentration of 1:1,000 up to 1:5,000 for 1 hour and finally detected by electrochemiluminescence (ECL; Bio-Rad, Hercules, CA, USA; Cat: 1705062).

The antibodies used were anti-HIF-1α (1:1,000, R&D Systems, Minneapolis, MN, USA; Cat: AF1935), anti-HIF-2α (1:1,000, R&D Systems, Minneapolis, MN, USA; Cat: AF2886), and anti-β-actin (1:5,000, Santa Cruz Biotechnology, Dallas, TX, USA) as described before (29). Molecular weight was determined using the Page Ruler™ protein ladder (Thermo Fisher Scientific, Waltham, MA, USA; Cat: 26616).

### Staining of THP-1 cells and FACS analysis

THP-1 cells (approx. 100,000 cells per measurement) were stained with the following antibodies (diluted 1:50): CD11a-PE-Cy7 (Cat: 301220), CD11b-PerCP (Cat: 101230), CD11c-BV421 (Cat: 371512), CX3CR1-BV421 (Cat: 341620), and PD-L1-APC (Cat: 329707) (all from BioLegend, San Diego, CA, USA). After 25-min staining in the dark, 650 µL RBC Lysis/Fixation Buffer (Cat: 422401; BioLegend, San Diego, CA, USA) was added to the samples and incubated for another 20 min. Subsequently, the suspension was centrifuged at 400 × *g* for 5 min, and the supernatant was discarded. The cell pellet was resuspended in 100 µL fresh PBS and used for Fluorescence-Activated Cell Sorting (FACS) analysis. Flow cytometry was performed using a MACSQuant 10 flow cytometer (Miltenyi Biotec, Bergisch-Gladbach, Germany), and data were analyzed using the FlowJo software version 10.0 (FlowJo, LLC, Ashland, OR, USA).

### Statistical analysis

GraphPad Prism Version 7.0f (GraphPad Software, Inc., San Diego, CA, USA) was used for Student’s t-tests or non-parametric Mann–Whitney test for pairwise statistical comparison of data. Correlation analyses were performed using multivariate regression with Pearson’s correlation coefficient. *p* < 0.05 (*), *p* < 0.01 (**), and *p* < 0.001 (***).

## Results

### Characterization of plasma-derived sEVs

**Figure 1:**
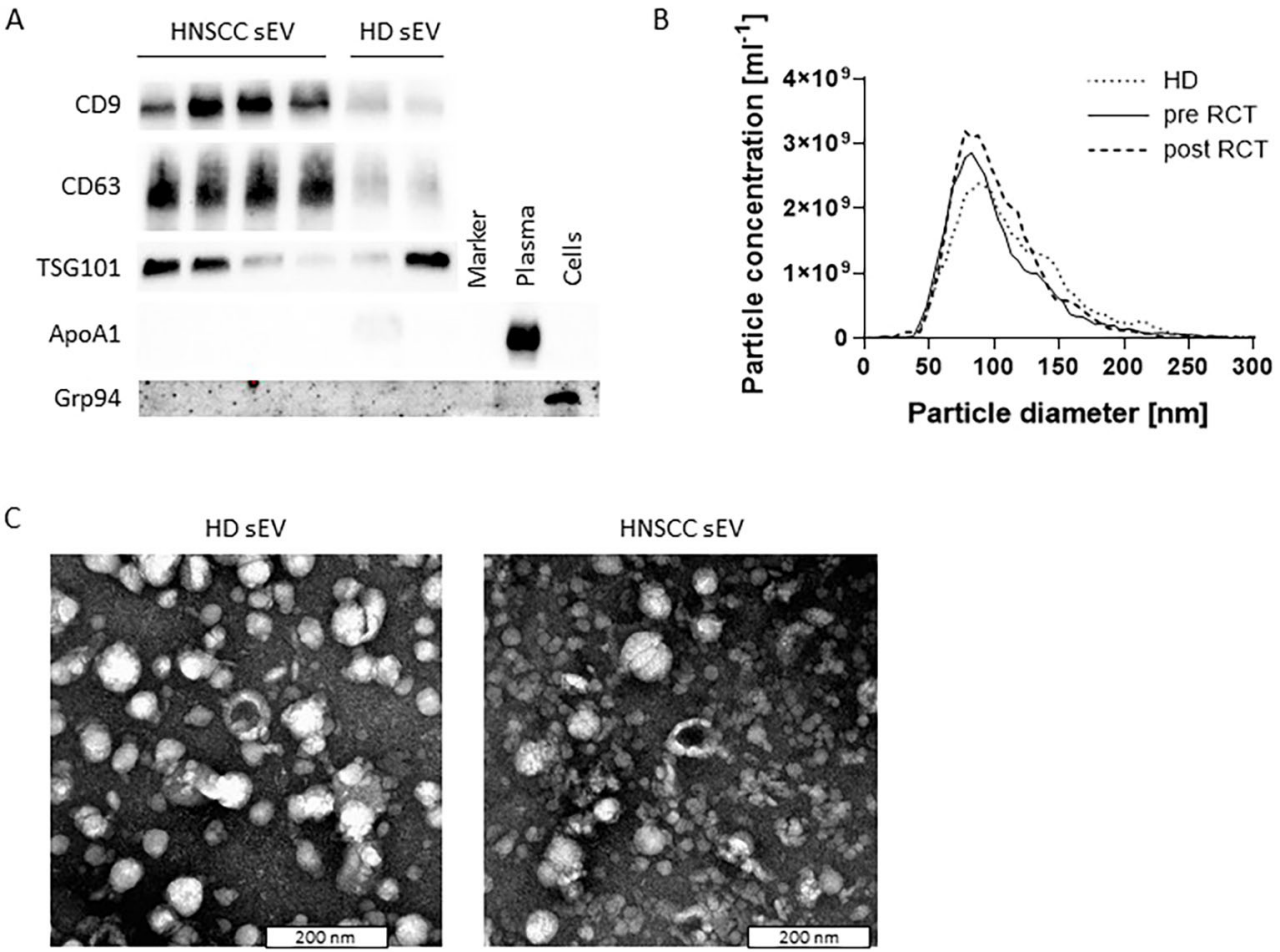
Characteristics of plasma-derived small extracellular vesicles (sEVs). **(A)** Representative Western blotting images from plasma-derived sEVs from healthy donors (HDs) and head and neck squamous cell carcinoma (HNSCC) patients before (pre) and after (post) radio/chemotherapeutic treatment (RCT). EV markers CD9, CD63, and TSG101 were present, while apolipoprotein ApoA1 and the cellular marker Grp94 were absent in EV preparations. Plasma and cells served as positive controls for ApoA1 and Grp94, respectively. **(B)** Representative sEV size distribution profiles determined via nanoparticle tracking analysis (NTA) revealed a size ranging from 50 to 250 nm with a median diameter of approximately 100 nm. **(C)** Representative sEV transmission electron microscopy (TEM) images revealed a vesicular shape; scale bar = 200 nm.

sEVs isolated from the plasma of HDs and HNSCC patients before (pre) and after (post) radio/chemotherapeutic treatment (RCT) were evaluated for protein composition via Western blotting analysis. Vesicles were positive for the tetraspanins CD63 and CD9 and the endosomal protein TSG101 and negative for the cellular protein Grp94 and apolipoprotein ApoA1 (**Figure 1A**). They showed a size ranging from 50 to 250 nm with a median diameter of approximately 100 nm (**Figure 1B**) and a vesicular shape in transmission electron microscopy (**Figure 1C**). The described results allow for the nomenclature of small EVs, in line with the MISEV 2023 recommendations (28).

**Figure 2:**
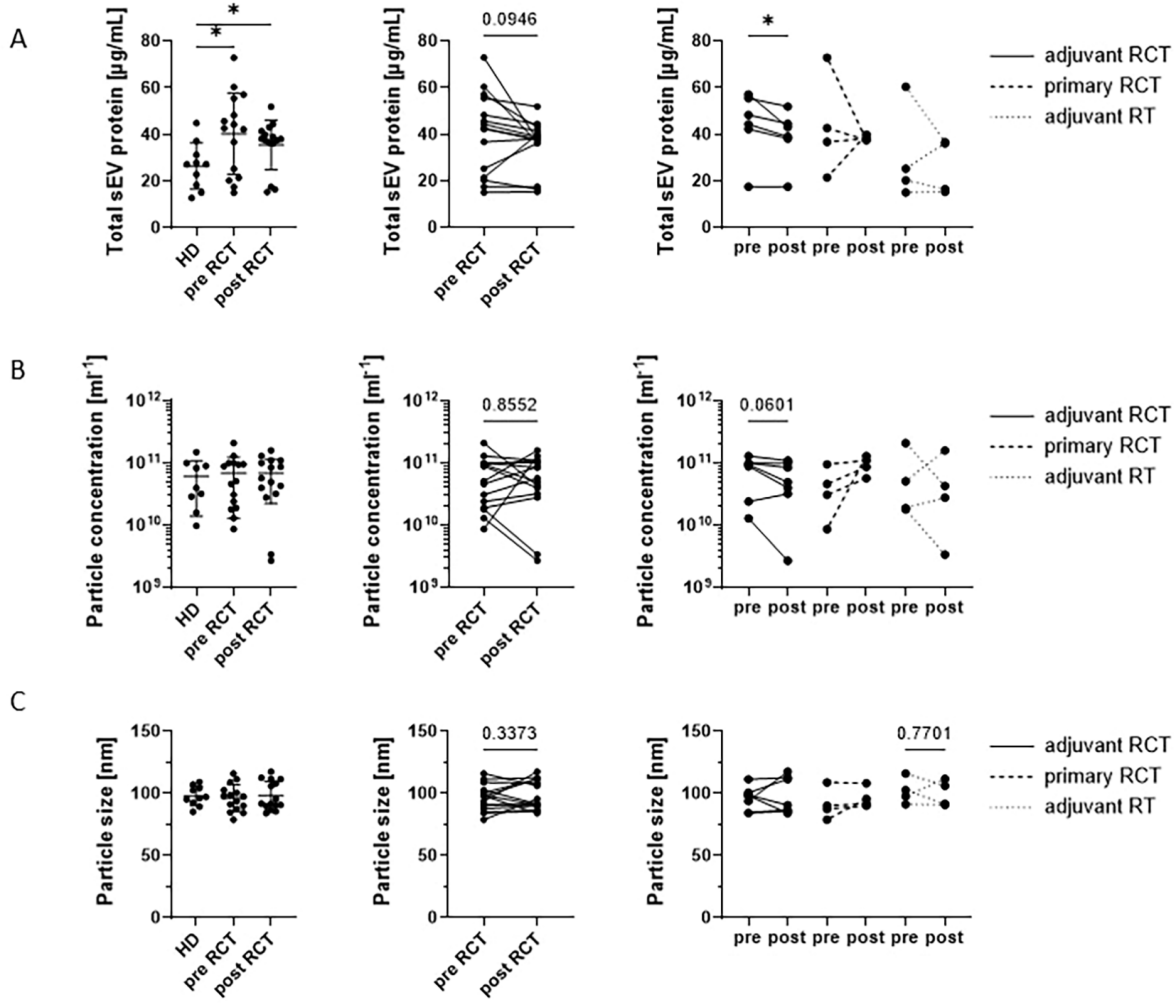
Characteristics of plasma-derived small extracellular vesicles (sEVs) comparing healthy donors (HDs) and head and neck squamous cell carcinoma (HNSCC) patients before (pre) and after (post) radio/chemotherapeutic treatment (RCT). Comparison of **(A)** total sEV protein, **(B)** particle concentration, and **(C)** particle size between HDs (n = 10) and HNSCC patients (n = 15) at the time points before and after treatment. In each subfigure, the left graph shows a comparison between HDs before RCT and after RCT, the middle graph compares paired pre- and post-RCT samples, and the right graph groups patients according to their individual therapy regimen [adjuvant RCT, primary RCT, and adjuvant radiotherapy (RT)]. Total sEV protein was significantly elevated in HNSCC patients, while no differences were observed for particle concentration and size. Furthermore, patients tended to have lower total sEV levels after RCT compared to the time point before RCT. Scatter plots show the mean with standard deviation (SD). *p < 0.05.

Total sEV protein, as an indirect estimate for sEV quantity, was significantly elevated in HNSCC patients both pre-RCT (FC 1.52) and post-RCT (FC 1.34) compared to HDs, while no differences were observed for particle concentration and size (**Figure 2A**, left). When comparing paired pre- and post-RCT samples, patients tended to have lower total sEV levels after RCT compared to the time point before RCT (FC 0.88, **Figure 2A**, middle). When comparing different treatment regimens, only patients receiving adjuvant RCT showed a significant reduction of total sEV protein after therapy (FC 0.88), compared to patients receiving primary RCT or adjuvant RT (**Figure 2A**, right). Although patients receiving adjuvant RCT also showed a trend toward reduced particle concentration after therapy, no other differences were observed for particle concentration and size (**Figures 2B, C**).

### Monocytic adhesion molecules

**Figure 3:**
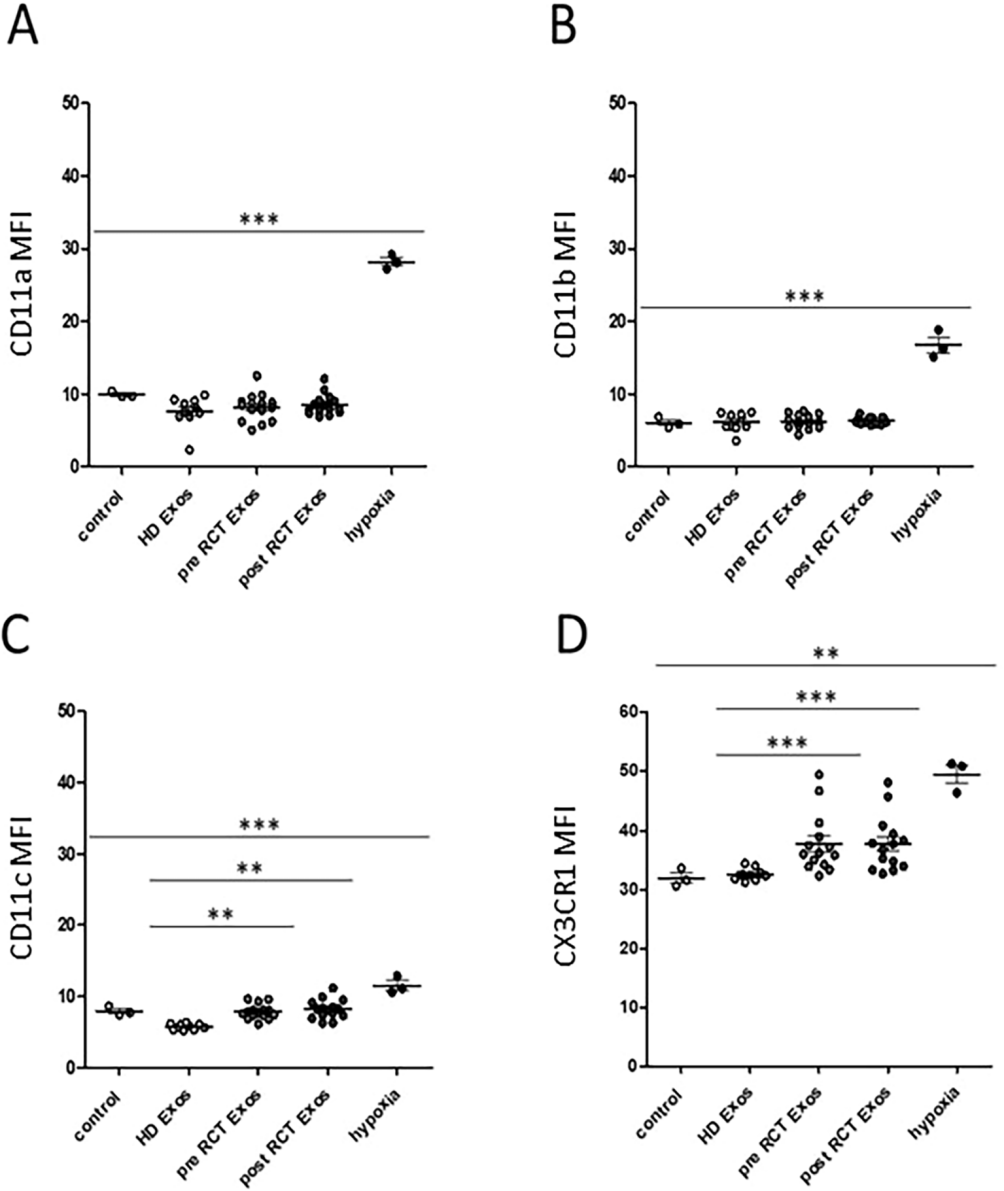
Flow cytometric analyses of monocytic adhesion molecules. Flow cytometric analysis revealed significantly increased expression levels of adhesion molecules **(A)** CD11a (*p* < 0.0001), **(B)** CD11b (*p* = 0.0008), **(C)** CD11c (*p* < 0.0001), and **(D)** CX3CR1 (*p* = 0.0006) upon 24 hours of hypoxic (5% O_2_) growth conditions compared to the normoxic controls. In addition, surface expression levels of adhesion molecules CD11c and CX3CR1 were significantly increased in response to plasma-derived small extracellular vesicles (sEVs) from head and neck squamous cell carcinoma (HNSCC) patients before [pre radio/chemotherapeutic treatment (RCT)] and after (post RCT), compared to sEVs from healthy donors (HDs). MFI, mean fluorescence intensity. **p < 0.01; ***p < 0.001.

Flow cytometric measurements of adhesion molecules CD11a, CD11b, CD11c, and CX3CR1 revealed significantly elevated expression levels after 24 hours of hypoxic (5% O_2_) growth conditions compared to the normoxic controls (**Figure 3**). Significantly increased expression of monocytic adhesion molecules CD11c and CX3CR1 could also be observed in response to an incubation with HNSCC plasma-derived sEVs compared to sEVs from HDs. In contrast, no differences were found between the pre- and post-RCT situations (**Figures 3C, D**).

**Figure 4:**
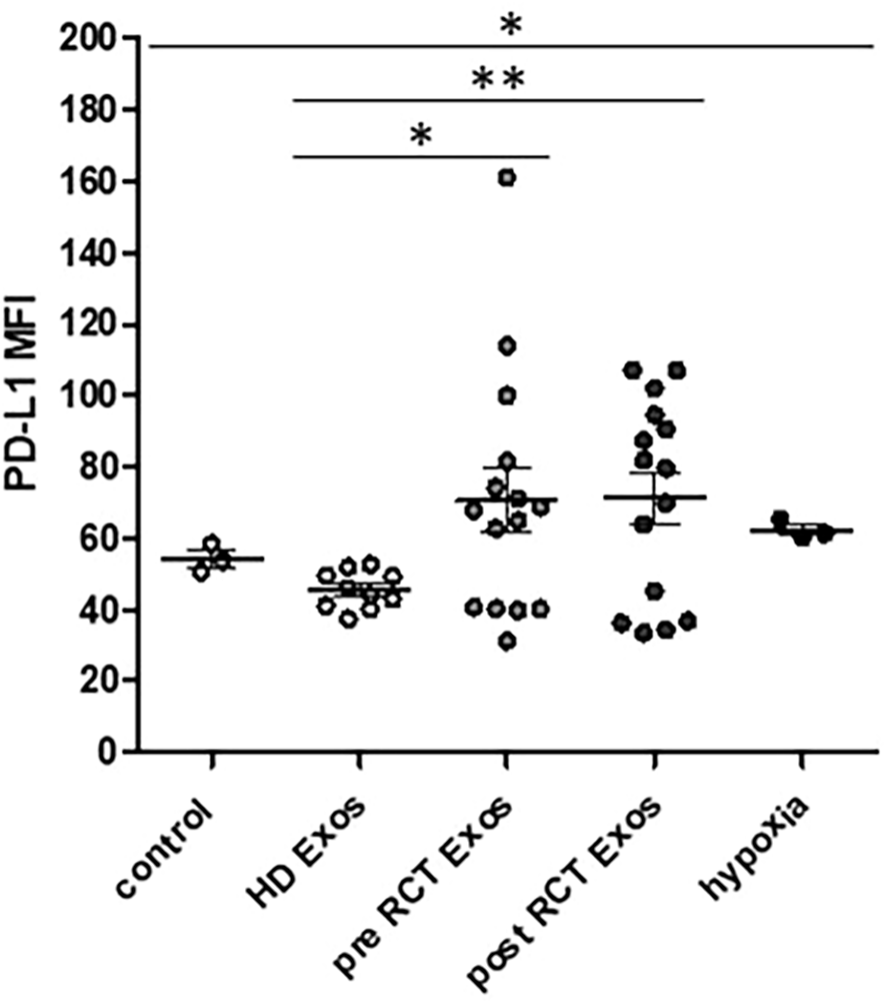
Flow cytometric analysis of checkpoint molecule. PD-L1 expression levels were significantly increased in response to hypoxia compared to the normoxic control. Furthermore, PD-L1 expression levels on THP-1 monocytes were significantly increased in response to plasma-derived small extracellular vesicles (sEVs) from head and neck squamous cell carcinoma (HNSCC) patients before (pre; *p* = 0.0320) and after (post; *p* = 0.0084) radio/chemotherapeutic treatment (RCT) compared to sEVs from healthy donors (HDs). *p < 0.05; **p < 0.01. MFI, mean fluorescence intensity.

Due to the known context of hypoxia and checkpoint molecule PD-L1, expression levels were measured on THP-1 monocytes in response to sEVs from healthy donors and HNSCC patients, as well as hypoxic growth conditions. Data revealed significantly increased PD-L1 expression levels on THP-1 monocytes in response to 24 hours of hypoxic growth conditions compared to the normoxic control and, furthermore, significantly increased monocytic PD-L1 levels upon incubation with plasma-derived sEVs from HNSCC patients (pre- and post-RCT) compared to sEVs from healthy donors (**Figure 4**).

**Figure 5:**
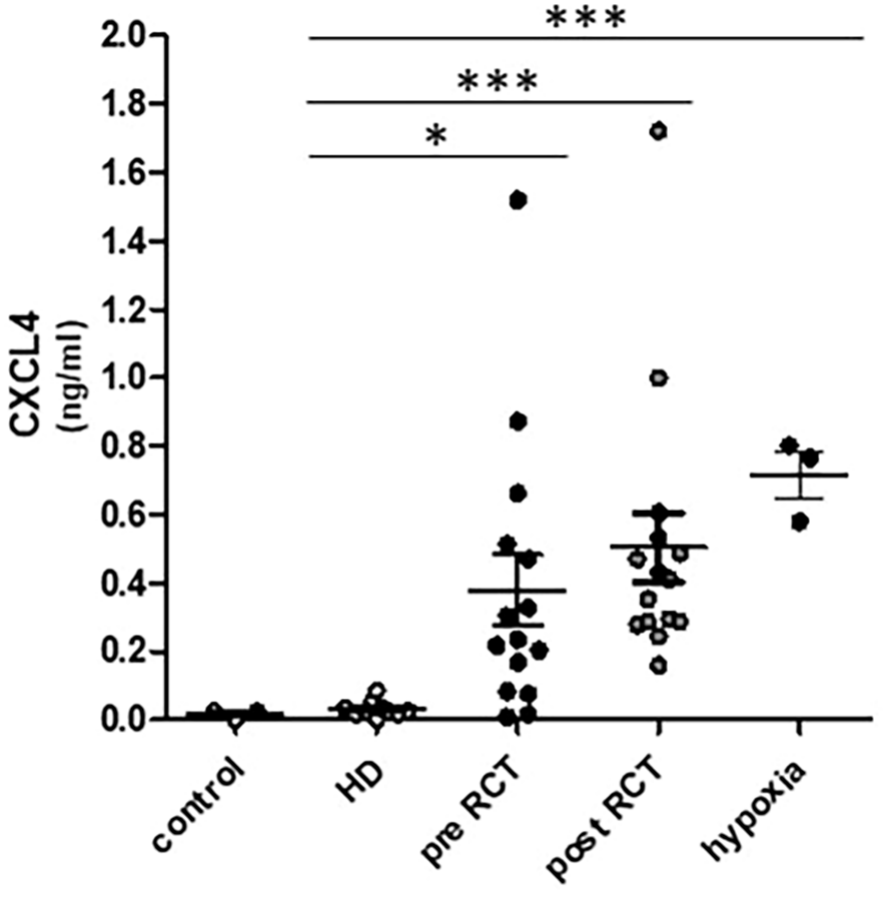
Enzyme-linked immunosorbent assay (ELISA) measurements of CXCL4 in cell culture supernatants. Data revealed significantly increased secretion levels of cytokine CXCL4 (ng/mL) by THP-1 in response to plasma-derived small extracellular vesicles (sEVs) from head and neck squamous cell carcinoma (HNSCC) patients before (pre; *p* = 0.0120) and after (post; *p* < 0.0001) radio/chemotherapeutic treatment (RCT) and hypoxic growth conditions (*p* < 0.0001) compared to sEVs from healthy donors (HDs). *p < 0.05; ***p < 0.001.

Moreover, ELISA measurements of cytokine CXCL4 (ng/mL) in cell culture supernatants revealed significantly increased levels of CXCL4 secretion by THP-1 monocytes in response to incubation with plasma-derived sEVs from HNSCC patients (pre- and post-RCT) compared to sEVs from healthy donors (**Figure 5**). Further correlation analyses revealed no significant correlations between monocytic PD-L1 expression and CXCL4 secretion in response to plasma-derived sEVs from HNSCC patients (pre- and post-RCT) (**Supplementary Figure 1**).

### HIF-2α accumulation upon HNSCC plasma-derived sEVs

To determine whether the observed induction of CXCL4 secretion in THP-1 monocytes may be connected with a sEV-driven regulation of hypoxia-related molecular pathways, the protein expression of HIF-1α and HIF-2α in monocytes was investigated upon incubation with plasma-derived sEVs from HNSCC patients and healthy donors using immunoblot analysis.

**Figure 6:**
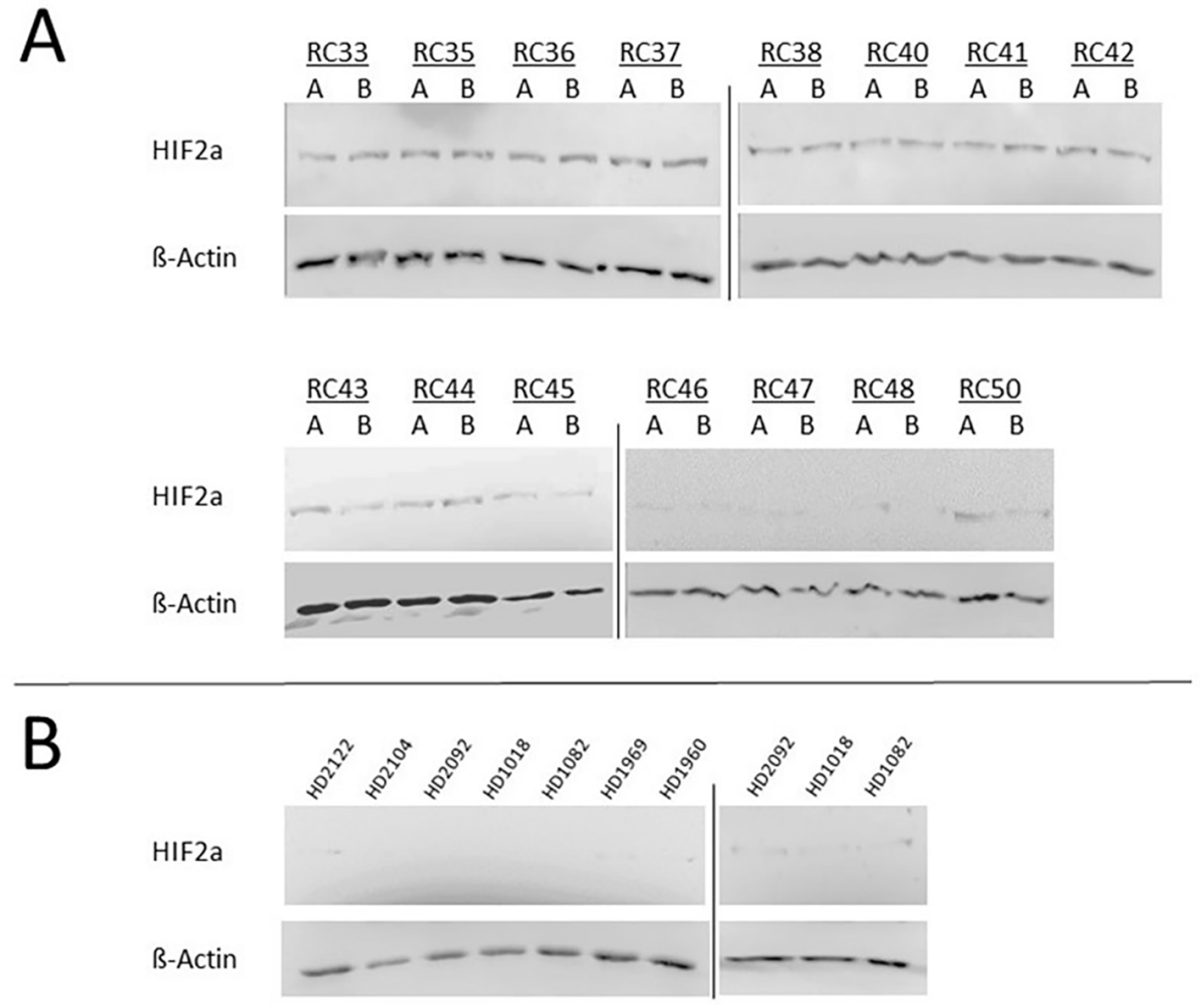
Immunoblot analysis of hypoxia-inducible factor (HIF)-2α protein levels in THP-1 monocytes in response to plasma-derived small extracellular vesicles (sEVs) from **(A)** head and neck squamous cell carcinoma (HNSCC) patients before (RC A) and after (RC B) radio/chemotherapeutic treatment and **(B)** from healthy donors (HDs). Data revealed a significant HIF-2α accumulation in THP-1 protein extracts upon 24 hours of incubation with plasma-derived sEVs from HNSCC patients before and after radio/chemotherapeutic treatment (RCT), whereas no HIF-2α protein was detectable in response to sEVs from healthy donors. Blots from different gels are delineated with dividing lines. β-Actin was used as a loading control.

Data revealed a significant HIF-2α accumulation in THP-1 protein extracts upon 24 hours of incubation with plasma-derived sEVs from HNSCC patients before and after RCT, whereas no HIF-2α protein was detectable in response to sEVs from healthy donors (**Figure 6**).

In contrast, the HIF-1α protein was not detectable in any of the analyzed protein extracts of THP-1 monocytes in response to the analyzed incubation conditions (data not shown).

### Profiling of hypoxia-related miRNAs of plasma-derived sEVs

Next, the presence and differential representation of 23 hypoxia-related miRNAs selected from the literature were analyzed in a cohort of 49 HDs and 80 HNSCC patients, who were comprehensively profiled for their sEV-miRNA cargo.

**Figure 7:**
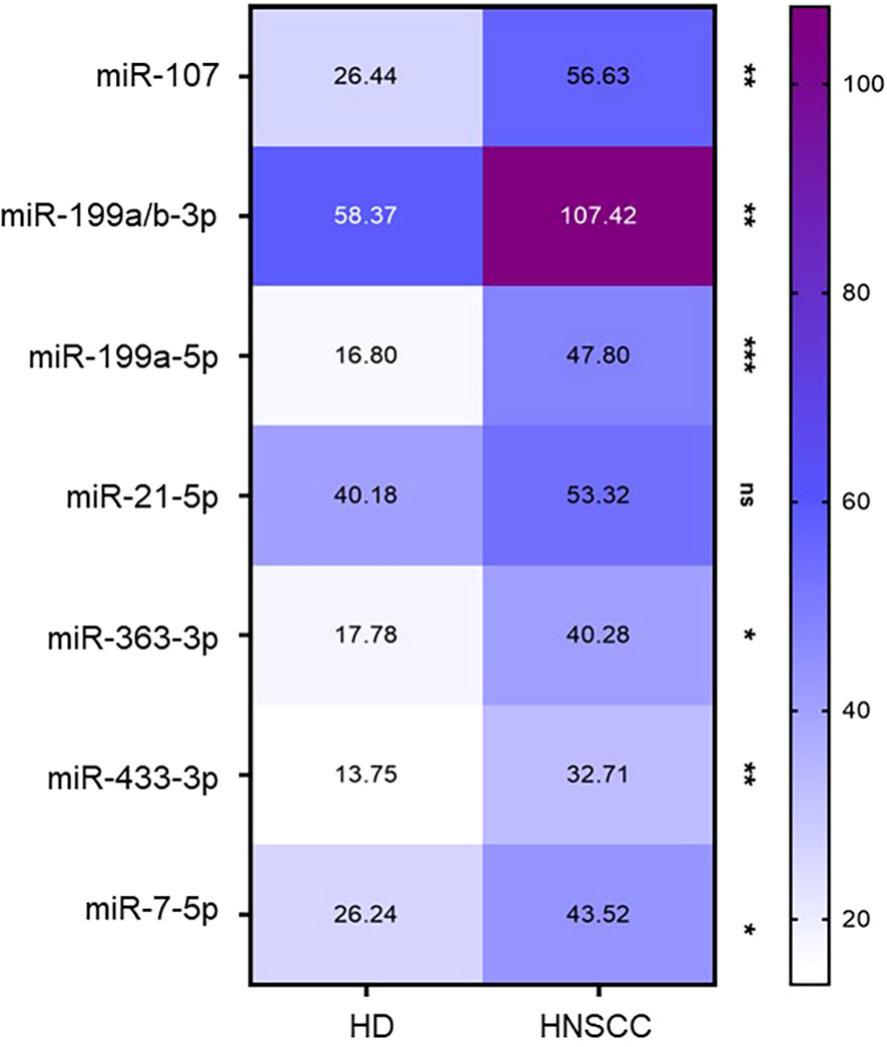
Hypoxia-related miRNAs in plasma-derived small extracellular vesicles (sEVs). Heatmap of hypoxia-related miRNAs showing the mean of normalized miRNA counts between healthy donors (HDs) (n = 49) and head and neck squamous cell carcinoma (HNSCC) patients (n = 80). Only those miRNAs present in our cohort are depicted. Ns, not significant; *p < 0.05; **p < 0.01; ***p < 0.001.

Of the selected 23 hypoxia-related miRNAs, seven were present in the sEV samples (**Figure 7**, **Supplementary Figure 2**). Of those, miRNAs 107, 199a/b-3p, 199a-5p, 363-3p, 433-3p, and 7-5p showed significantly elevated levels in sEVs of HNSCC patients compared to sEVs of HDs (**Figure 7**).

## Discussion

Hypoxia is a common feature of the tumor microenvironment (TME) of most solid HNSCC and contributes to increased metastasis, higher recurrence rates, and therapy resistance (30). Furthermore, hypoxia is associated with immune disturbances and the regulation of signaling pathways, including autophagy, metabolism, and cell stress (31–35).

HIF-1α and HIF-2α are widely expressed in different innate immune cells and are the key regulators to facilitate the cellular response to hypoxic conditions in different inflammatory and malignant diseases (36–40), whereas the recruitment of these transcriptional coactivators is prevented under normoxic conditions (41–44).

Investigations on HIF-1α and HIF-2α should take into account that both transcriptional activators have different stabilities and separate underlying regulation systems (45, 46). Despite having similar structures and functional overlap, both HIF isoforms have been shown to reveal distinct expression patterns and functions at the cellular level in monocytes and macrophages (47). HIF-1α is rapidly stabilized under acute hypoxia and induced by Th1 cytokines in M1 macrophage polarization, whereas HIF-2α regulates cell responses to chronic hypoxia and is induced by Th2 cytokines during an M2 response (48–51).

In addition to a wide variety of soluble mediators such as cytokines and chemokines, sEVs are closely associated with the regulation of hypoxic processes within the TME and the systemic circulation of tumor patients (52–56).

It has been shown that sEV-related cargo transfer is more frequent in a hypoxic TME and that HIF-1α is involved in the biogenesis and composition of sEVs in HNSCC (57). Plasma-derived sEVs are well-established messengers of intercellular communication and promising bioliquid indicators for treatment response and tumor progression in head and neck cancer (6). For instance, checkpoint molecule PD-L1-positive plasma-derived sEVs from different cell types, including tumor cells, T cells, B cells, and monocytes, have been shown to support an immune evasion in head and neck cancer patients (5, 58).

Our data revealed comparable influences of plasma-derived sEVs from HNSCC patients and hypoxic growth conditions on expression profiles of different adhesion molecules and checkpoint molecule PD-L1 in human THP-1 monocytes.

Monocytic cell lines are established for *in vitro* investigations such as macrophage biology and polarization, although they are certainly a simplified experimental model of the *in vivo* situation and display certain differences compared to primary human macrophages (59–61). In addition, monocytic cell lines ensure a homogeneous genetic background and minimize the variability in the phenotypes of monocyte-derived macrophages (19, 62).

Furthermore, significantly elevated secretion of hypoxia-associated chemokine CXCL4 was observed in response to plasma-derived sEVs from HNSCC patients compared to sEVs from healthy donors. Chemokine CXCL4 has been shown to promote the differentiation of monocytes into myeloid-derived suppressor cells (MDSCs), thus supporting T-cell inhibition and tumor metastasis (63, 64). While we used a combination of differential centrifugation, ultrafiltration, and ultracentrifugation to isolate EVs, we acknowledge that density gradients or SEC can further increase EV purity. However, given the methodological stringency, the EV controls, and our consistent group-wise processing, we consider the impact on biological interpretation to be minimal.

Of note, immunoblots of monocytes incubated with sEVs from HNSCC patients do not detect increased expression patterns of ubiquitous HIF-1α but increased expression of the more restricted/tissue-specific HIF-2α. HIFs become rapidly degraded under physiological O_2_ concentrations and stabilized in response to hypoxic conditions and then translocate into the nucleus to activate numerous hypoxia-associated target genes (65, 66).

It has been shown in endothelial cells that HIF-1α is involved in the initial adaptation to hypoxic stress, while HIF-2α expression starts rather later upon prolonged hypoxia. Basically, different studies have emphasized the importance of balanced HIF-1α/HIF-2α protein abundances and imply different physiological functions of these two transcriptional activators (67). In this context, human transcription factor Spliceosome-Associated Factor 1 (SART1) has been proposed as a crucial mediator of a controlled HIF-1α destabilization in coordination with a HIF-2α stabilization in the course of the hypoxic process (68, 69).

The main way of sEV-mediated modulation of recipient cells is not a direct protein transfer but rather a transfer of specific miRNA molecules (13, 14, 70). MiRNAs are non-coding, single-stranded RNAs that are post-transcriptional regulators of numerous biological pathways, including the modulation of HIF expression and signaling (71, 72).

Although many different hypoxia-associated miRNAs have been identified, a precise functional role has been found only for a limited number of these molecules (73–75).

In this study, miRNA profiling was used to analyze the differential abundances of 23 hypoxia-related miRNAs in a cohort of 49 HDs and 80 HNSCC patients. Measurements identified six significantly elevated hypoxia-associated miRNAs (miRs 107, 199a/b-3p, 199a-5p, 363-3p, 433-3p, and 7-5p) in sEVs of HNSCC patients compared to sEVs of HDs.

**Figure 8:**
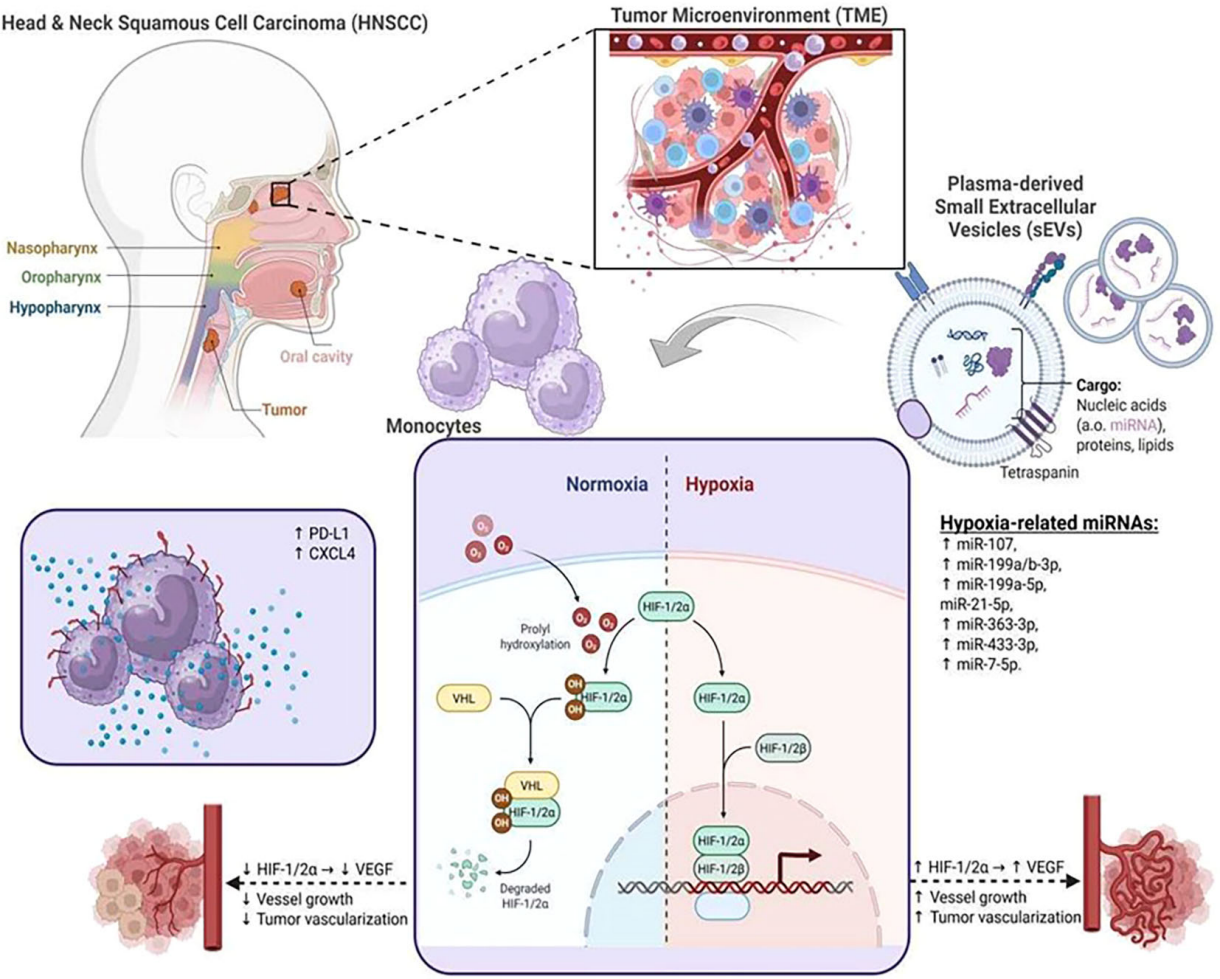
Graphical presentation of the potential regulatory impact of hypoxia-related miRNAs in plasma-derived small extracellular vesicles (sEVs) from head and neck squamous cell carcinoma (HNSCC) patients. Hypoxia is an important hallmark of the tumor microenvironment (TME) in solid tumors, in which tumor-associated plasma-derived sEVs have gained increasing attention as important regulators. Transfer of plasma-derived sEVs from HNSCC patients stimulated increased levels of checkpoint molecule PD-L1 and chemokine CXCL4 secretion in human monocytes. An accumulation of hypoxia-inducible factor (HIF)-2α was associated with hypoxia-regulating miRNAs in sEVs from HNSCC patients, suggesting a potential tumor-associated systemic sEV-miRNA-mediated hypoxia transfer. This figure was created with BioRender.com.

The graphical illustration in **Figure 8** serves as a working model of these findings in the context of the potentially underlying regulatory network (**Figure 8**).

Among the identified miRNA molecules, miRNA107, miRNA199, and miRNA433 have been suggested to directly target HIF-1α and thus may contribute to the described HIF switch and increased HIF-2α levels (76–78). More precisely, we identified distinct miRNA199 subtypes (199a/b-3p, 199a-5p), and it has previously been described that polymorphisms in the miRNA199 target site within the HIF-1α mRNA sequence are linked to an increased pancreatic carcinoma risk (79). MiRNA363-3p has been identified as a HIF-dependent miRNA and has recently been shown to induce chemoresistance in breast cancer (80). Furthermore, miRNA profiling revealed significantly increased abundances of the HIF-2α-specific miRNA 7-5p (81), which is associated with different pathological processes, including proliferation, migration, and metastasis, as either a tumor suppressor (82, 83) or a tumor promoter (84, 85). For instance, increased resistance to radiation in response to an overexpression of miR-7-5p has been shown in different clinically relevant cancer cell lines (86).

Our data emphasize the importance of ongoing comprehensive investigations regarding specific gain-of-function or loss-of-function experiments on the proposed miRNA-HIF-2α axis and the meaningfulness of a systemic sEV-miRNA mediated hypoxia transfer to circulating immune cells for therapy response and the individual course of the disease.

## Data Availability

The datasets presented in this study can be found in online repositories. The names of the repository/repositories and accession number(s) can be found in the article/**Supplementary Material**.

## Supplementary material

Supplementary Figure 1 Correlation analyses revealed no significant correlations between monocytic PD-L1 expression and CXCL4 secretion in response to plasma derived sEVs from **(A)** HNSCC patients before (pre RCT) or **(B)** HNSCC patients after (post RCT) radio/chemotherapeutic treatment. The correlation coefficient (r) and p values are given for each correlation. p < 0.05 was considered as significant. MFI: mean fluorescence intensity.
