# Supplementary figures and images for: HIF-2α accumulation in human monocytes upon transfer of hypoxia-associated miRNAs via plasma-derived small extracellular vesicles from head and neck cancer patients
Source: Front Oncol. 2026 Jan 12;15:1701388. doi: 10.3389/fonc.2025.1701388 (PMC12833699; doi:10.3389/fonc.2025.1701388)

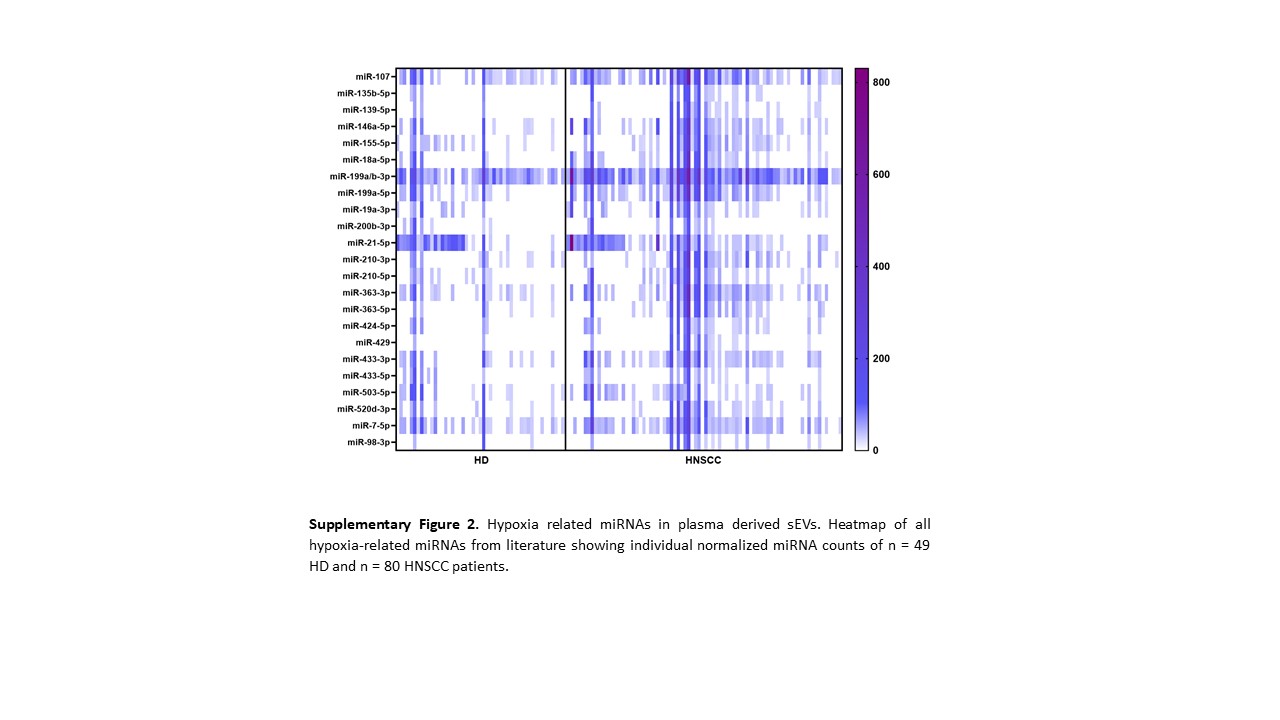

Supplement: Supplementary Figure 1 — Correlation analyses revealed no significant correlations between monocytic PD-L1 expression and CXCL4 secretion in response to plasma derived sEVs from (A) HNSCC patients before (pre RCT) or (B) HNSCC patients after (post RCT) radio/chemotherapeutic treatment. The correlation coefficient (r) and p values are given for each correlation. p < 0.05 was considered as significant. MFI: mean fluorescence intensity. [file Image1.jpeg]

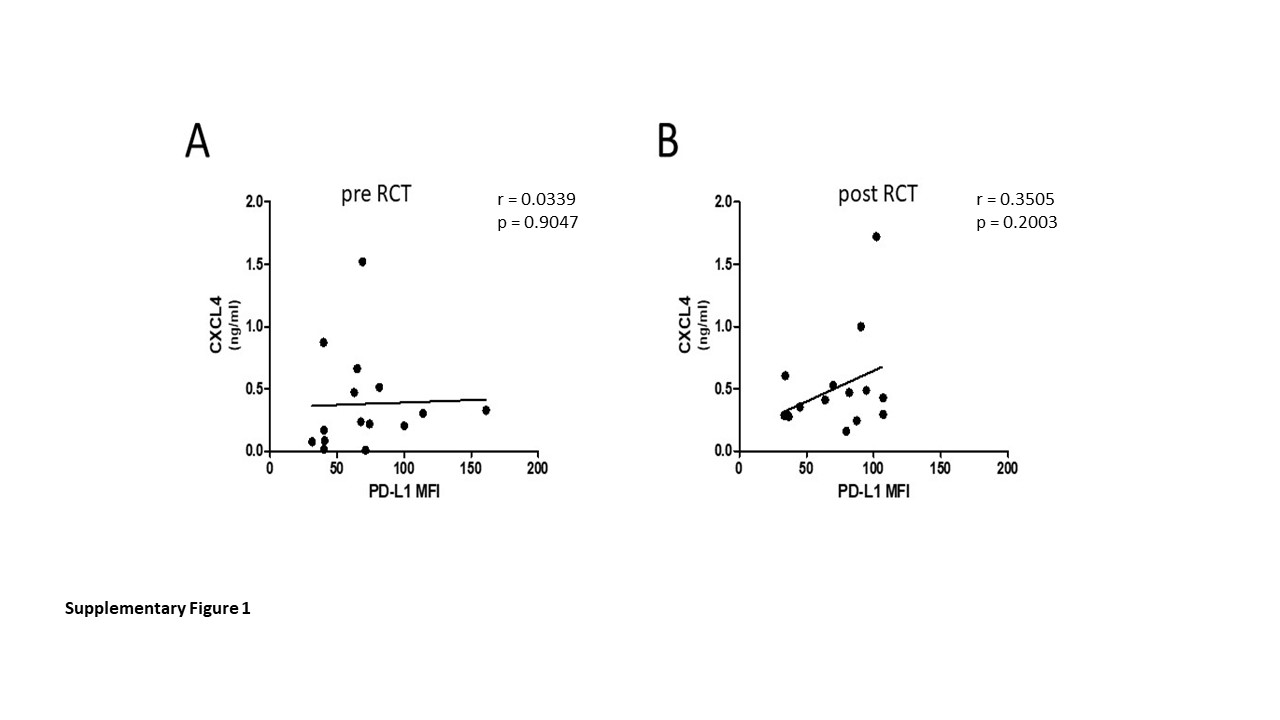

Supplement: Supplementary file 2 [file Image2.jpeg]
